# Use and Disuse of Technology Among Persons With Memory Concerns and Their Caregivers During the COVID-19 Pandemic

**DOI:** 10.1093/geroni/igab046.843

**Published:** 2021-12-17

**Authors:** Ashley Millenbah, Elizabeth Albers, Jude Mikal, Jessica Finlay, Eric Jutkowitz, Lauren Mitchell, Brenna Horn, Joseph Gaugler

**Affiliations:** 1 University of Minnesota, University of Minnesota, Minnesota, United States; 2 University of Minnesota, Minneapolis, Minnesota, United States; 3 University of Michigan, Ann Arbor, Michigan, United States; 4 Brown University, Brown University, Rhode Island, United States; 5 Emmanuel College, Boston, Massachusetts, United States; 6 University of Minnesota/ Families and Long-Term Care Projects, Minneapolis, Minnesota, United States

## Abstract

Social distancing and shelter-in-place orders designed to curb the spread of COVID-19 increased isolation among persons with memory concerns (PWMC) and increased the burden on individuals providing their care. Technology, such as smartphones or tablets, has demonstrated potential to improve the social connections and mental health of older adults; however, older adults historically have been reluctant to adopt new technology. We aimed to understand why some PWMC and their caregivers used new technology to adapt to lifestyle changes during the COVID-19 pandemic while others did not. In this study, we used data collected in 20 qualitative interviews from June to August, 2020 with PWMC and their family caregivers to assess changes in and barriers to technology use. Qualitative thematic analysis identified three themes which explained motivations for using new technology during a pandemic: 1) seeking relief from caregiver burden, 2) alleviating boredom, and 3) maintaining social connection. Results further revealed lingering barriers to PWMC and caregiver adoption of technologies, including: 1) PWMC dependence upon caregivers, 2) a lack of familiarity with technology, and 3) difficulties using technology. This in-depth investigation suggests that technology has the ability to provide caregivers relief from caregiving duties and provide PWMC with more independence during periods of pronounced isolation

